# Zigbee-Based Wireless Sensor Network of MEMS Accelerometers for Pavement Monitoring †

**DOI:** 10.3390/s24196487

**Published:** 2024-10-09

**Authors:** Nicky Andre Prabatama, Mai Lan Nguyen, Pierre Hornych, Stefano Mariani, Jean-Marc Laheurte

**Affiliations:** 1 Univ Gustave Eiffel, CNRS, ESYCOM, F-77454 Marne-la-Vallée, France; jean-marc.laheurte@univ-eiffel.fr; 2 Univ Gustave Eiffel, MAST-LAMES, F-44344 Bouguenais, France; 3 Department of Civil and Environmental Engineering, Politecnico di Milano, 20133 Milano, Italy; stefano.mariani@polimi.it

**Keywords:** pavement monitoring, Zigbee, wireless sensor network, accelerometers

## Abstract

In this paper, we propose a wireless sensor network for pavement health monitoring exploiting the Zigbee technology. Accelerometers are adopted to measure local accelerations linked to pavement vibrations, which are then converted into displacements by a signal processing algorithm. Each device consists of an on-board unit buried in the roadway and a roadside unit. The on-board unit comprises a microcontroller, an accelerometer and a Zigbee module that transfers acceleration data wirelessly to the roadside unit. The roadside unit consists of a Raspberry Pi, a Zigbee module and a USB Zigbee adapter. Laboratory tests were conducted using a vibration table and with three different accelerometers, to assess the system capability. A typical displacement signal from a five-axle truck was applied to the vibration table with two different displacement peaks, allowing for two different vehicle speeds. The prototyped system was then encapsulated in PVC packaging, deployed and tested in a real-life road situation with a fatigue carousel featuring rotating truck axles. The laboratory and on-road measurements show that displacements can be estimated with an accuracy equivalent to that of a reference sensor.

## 1. Introduction

Poorly maintained or damaged pavements can cause road accidents and reduce the efficiency of freight transport. However, detecting pavement deterioration before it is too late to repair remains a difficult and costly task. A relevant solution is sensor-based monitoring of the health of pavements, to assess their condition and take actions for their maintenance, if necessary, based on any signs of deterioration.

Numerous pavement-monitoring technologies already exist, but most of them are still very expensive. In [1], the costs of five different pavement-monitoring systems were compared and shown to range from USD 23,500 to USD 131,300 per lane. These high costs are mainly due to the fact that most pavement monitoring systems use power and data cables, requiring a trench to connect the sensors in the pavement to the acquisition unit located at the roadside. As a result, roadside monitoring solutions based on wireless, cable-free devices like that presented in this work can become affordable and considerably more cost-effective than wired solutions, both in relation to their installation and their maintenance.

Monitoring methodologies generally involve the measurement of pavement strain or (vertical) displacement through geophones, strain sensors or accelerometers. Strain sensors [2,3,4,5] are used to measure the transverse or longitudinal strain components of the pavement layer, with the data being generally saved to and logged in the roadside unit. In the particular case of the configuration presented in [3,5], the data were transmitted continuously to the cloud via an internet connection. Other devices, such as those described in [6,7] rely on geophones to measure road vibrations, while in [6,8,9,10,11,12], accelerometers were adopted. For configurations using geophones or accelerometers, the conversion to pavement displacement can be performed with a time integration procedure, such as that presented in [6,12], to remove rid artifacts linked to measurement noise and ensure that results are objective as far as the pavement’s response to the external actions is concerned.

The world is full of wireless sensor networks (WSNs), wherein data exchange between devices can be implemented easily without the use of cables, and pavement monitoring is no exception. The selection of the most appropriate wireless communication protocol (like, e.g., WiFi, Zigbee, BLE and LoRa) depends on the characteristics of the targeted application in terms of energy consumption, data rate and communication range. For example, a network using the WIFI protocol was proposed in [1,13,14]. The system consisted of acceleration sensors, a microcontroller and a battery packaged in a hard plastic case filled with fused silica. Another WSN for pavement instrumentation based on the LoRa protocol was presented in [15], which was based on acceleration measurements. In both cases, the acceleration-sensing node performed raw data acquisition, processing to extract characteristic features, store them and transmit them to the gateway. In the configuration explained in [16], a MicroLab MRF49XA transceiver (Microchip Technology, Chandler, AZ, USA) operating at 433 MHz was used with an accelerometer. The electronics were integrated into a cylindrical housing made of acrylic glass (Perspex), and the integrated device could be programmed wirelessly. The configuration presented in [2] consists of a wireless strain sensor with a Bluetooth transceiver. The microcontroller’s 10 bit Analog to Digital Converter (ADC) acquires the strain gauge signal, translates it into a Universal Asynchronous Receiver–Transmitter (UART) protocol and sends it to the data acquisition card using the Bluetooth protocol. The system was also equipped with a wireless energy harvesting system, used to charge a supercapacitor when the measurement was not performed.

In this work, we extend the variety of tests presented in [17] to fully assess the capacity and performance of the proposed monitoring strategy. New laboratory tests are carried out to account for different truck speeds and travel peaks and, further than that, a field test is carried out on a real roadway. Micro-electromechanical systems (MEMS) accelerometers are chosen in place of others, because of their ease of integration and low costs. As mentioned in [14], since roadway vibrations cause accelerations ranging from 0.09 m/s^2^ to around 1.9 m/s^2^, an accelerometer with a sensitivity of up to 2.25 m/s^2^ and a bandwidth of 50 Hz is sufficient to capture the effects of vehicles’ axles on the roadway. On the other hand, the pavement displacement induced by traffic can usually range between 0.1 mm and 1 mm [18]. The prototype described in Section 2 must therefore be able to operate within the aforementioned ranges of acceleration or displacement as well as bandwidth. The on-board unit is developed using an ESP32 Pico Kit microcontroller (Espressif Systems, Shanghai, China), together with an accelerometer and a Zigbee module to obtain and transmit acceleration data wirelessly. For the roadside unit, a Raspberry Pi 4 (Cambridge, UK) with a Zigbee module is used to receive wireless acceleration data and store it in a csv file.

In Section 3, three different commercial off-the-shelf accelerometers are tested in the laboratory, using a vibrating pot and by applying a sinusoidal low acceleration signal with an ad hoc defined frequency. This test enables a dynamic calibration to be carried out, so that the characteristics of the three accelerometers, such as sensitivity and power consumption, are obtained. A second laboratory test is carried out using a vibration table by applying displacement signals typical of a five-axle truck and featuring different downward displacement peaks and speeds. Dedicated signal processing is next adopted to convert accelerations into displacements. The obtained time histories are compared with the output of a reference sensor available on the vibration table. An error indicator calculation proves that the proposed solution is capable of detecting the pavement displacement induced by a five-axle truck and characterized by different amplitudes and speeds. In Section 4, a preliminary test on a real road is also carried out, placing the sensor on the road and using a fatigue carousel to induce the vibrations of truck axles in the surrounding pavement. These additional results show that the device is capable of accurately detecting the acceleration induced by passing truck axles.

## 2. System Architecture

**Figure 1:**
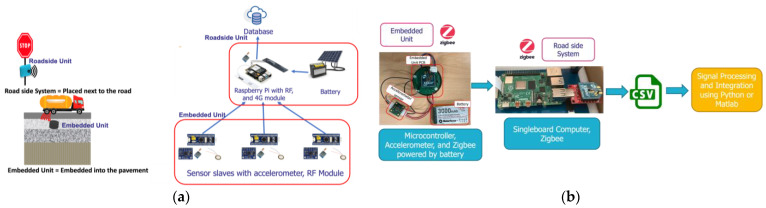
(**a**) System Architecture; (**b**) prototype tested in the laboratory.

The system architecture (Figure 1a) and the assembled prototype (Figure 1b) are based on a slave (on-board unit integrated into the pavement) and a master (roadside unit) that communicate wirelessly using the Zigbee wireless communication protocol. The on-board unit measures the acceleration resulting from pavement vibrations, and sends the data to the roadside unit. The Zigbee technology is chosen because it consumes little energy in transmission mode, with a data rate and communication range sufficient for the proposed application.

**Figure 2:**
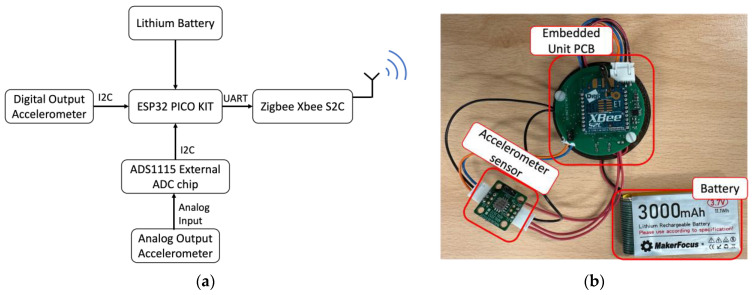
(**a**) Block diagram of the embedded unit; (**b**) embedded unit prototype.

The block diagram and a picture of the on-board unit are, respectively, shown in Figure 2a,b. The on-board unit consists of an accelerometer, an ESP32 Pico D4 microcontroller (Espressif Systems, Shanghai, China), an ADS1115 (Texas Instrument, Dallas, TX, USA) external ADC chip and an XBee S2C Zigbee (DIGI, Hopkins, MN, USA) module, all powered by a Makerfocus (Shenzhen, China) 3000 mAh battery. An external ADC chip ADS1115 is also added to the system to obtain the analog output of the accelerometer, as it was observed that the microcontroller has a non-linear ADC input. The Xbee S2C module for the receiver and transmitter is configured using XCTU software version 6.5.13 to set the appropriate address for the coordinator and end node, as well as the same baud rate between the two modules. If the address is not configured correctly and the baud rate is not the same, there will be no communication between the two modules. The microcontroller communicates with the Xbee S2C module using the UART protocol. The Zigbee Xbee S2C contains a wired antenna operating in the 2.4 GHz frequency band. Three different accelerometers are tested, i.e., ADXL355, ADXL354 and MS1002. Since it has a digital output, the ADXL355 accelerometer is connected directly to the microcontroller via I2C by means of an I2C jumper. Conversely, an external ADC chip ADS1115 must be connected to the analog outputs of the ADXL354 or MS1002 via an AIN jumper. The microcontroller is programmed from a PC using the CP2102N programming chip via the microUSB port. The ESP32 Pico D4 microcontroller obtains the acceleration data from the accelerometer and converts it into appropriate user data, then sends it to the Xbee S2C module using the UART protocol. The Xbee S2C module then transmits the data wirelessly to the roadside unit. The voltage required to power the on-board unit is a minimum of 3 V, up to a maximum of 3.6 V. The 3000 mAh battery is connected to a battery connector on the PCB and provides power through an LDO AMS1117 regulator, which supplies 3.3 V to power all components. The system’s programmed data rate during vibration table and pavement testing ranges from 70 to 130 Hz.

With the chosen system architecture and the components used, the main limitation is the lifetime of the on-board unit. The total power consumption is 67.8 mA in active mode and 1.2 mA in standby mode. This gives a device lifetime of 66 days for a configuration where the device is only used for 15 min a day to take measurements.

**Figure 3:**
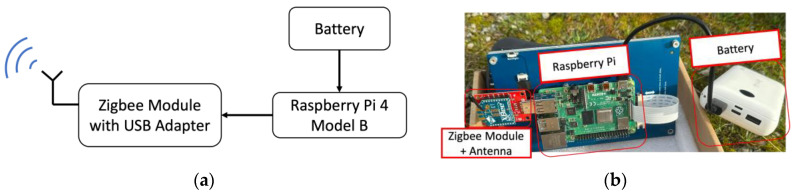
(**a**) Block diagram of the roadside unit; (**b**) roadside unit system.

For the roadside unit, a Raspberry Pi 4 with an Xbee S2C module and a USB adapter for the Zigbee module are used to receive wireless acceleration data. The block diagram and a picture of the roadside unit are reported in Figure 3a,b. A custom Python code was programmed to receive the acceleration data from the on-board unit and save them in a csv file. Tracing and signal processing can then be carried out in this csv file. The signal processing performed to obtain the displacement is also located in the roadside unit. The roadside unit is powered using a 20,000 mAh power bank battery. A mini screen displays the Raspberry Pi graphical user interface (GUI) and controls the measurement process.

## 3. Laboratory Tests with Vibrating Pot and Vibrating Table

### 3.1. Description of the Setups

In order to assess the performance and accuracy of the proposed system before its application in the real world, two different laboratory tests are carried out using a vibration pot and a vibration table. Three different MEMS accelerometers are chosen for the laboratory evaluation with the developed on-board unit: ADXL355 (Analog Devices, Wilmington, MA, USA), ADXL354 (Analog Devices) and MS1002 (Safran Colibrys, Yverdon-les-Bains, Switzerland). The three accelerometers have different data rates depending on several factors, such as microcontroller processing time and sensor data acquisition time (70 Hz for the MS1002, 70 Hz for the ADXL354 and 80 Hz for the ADXL355). The aim of the vibration pot test is to carry out a dynamic calibration and obtain the main characteristics of the accelerometers, such as sensitivity, noise and power consumption. The test is performed by applying a low sinusoidal acceleration with an ad hoc defined frequency.

**Figure 4:**
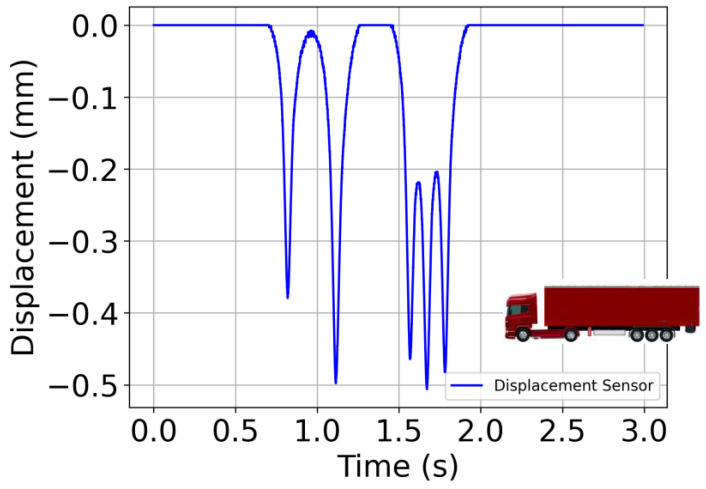
Five-axle truck displacement signals used for the vibrating table.

**Figure 5:**
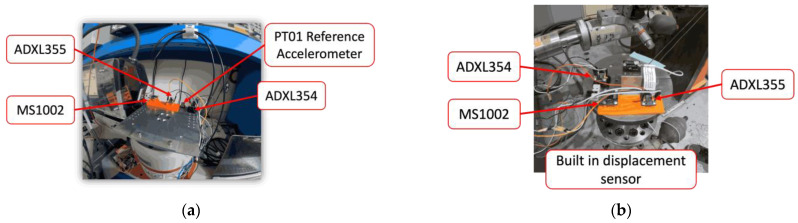
(**a**) Vibrating pot test; (**b**) vibrating table test.

The second test is carried out with a vibration table. The applied signal is typical of a five-axle truck, as depicted in Figure 4, and is tuned by way of the maximum displacement amplitudes and truck speed. A Messotron LVDT WLC 100 (Messotron, Seeheim-Jugenheim, Germany) displacement transducer integrated into the vibration table is used as a reference transducer. The test is performed with two different maximum displacement peaks, namely 0.25 mm and 0.5 mm, at a vehicle speed of 45 km/h, and for two additional truck speeds, namely 18 km/h and 92 km/h, with a displacement peak of 0.25 mm. The speed limits for trucks in various European countries are listed in [19,20,21]. Almost all countries impose a speed limit of 80 km/h to 90 km/h on motorways. On the other hand, according to the speed measurement data presented in [22], many trucks still drive above the speed limit, e.g., 87% in Sweden, 20% in Cyprus and 16% in Ireland. This is the main reason why a speed of 92 km/h was chosen to mimic trucks exceeding the speed limit. In urban areas, 95% of European countries limit speed to 50 km/h, while several countries apply a lower speed limit, e.g., 48 km/h in the UK, 40 km/h in Malta and Bulgaria, as reported in [19,20,21]. Thus, 45 km/h was used for the average speed on urban roads. In [19,20,21] it is stated that several countries apply special rules for truck speed limits in residential areas, e.g., Belgium, Ukraine and Belarus, which limit speed to 20 km/h in residential areas, and Slovenia, which limits speed to 10 km/h in areas with many pedestrians. This is why 18 km/h has been chosen as a reasonable speed limit for residential areas. The configurations of the vibrating pot and vibrating table tests are, respectively, illustrated in Figure 5a,b.

### 3.2. Results Obtained with the Vibrating Pot

Data related to the sensitivity, power consumption and noise of the three MEMS accelerometers, as measured during the vibrating pot tests, are shown in Table 1. It can be seen that the ADXL354, with 155.2 μA, has the lowest power consumption among the considered accelerometers. The noise of the three MEMS accelerometers was obtained under no-motion conditions during testing; as proposed in [23], the standard deviation of measurements is extracted from these no-motion signals as:

(1) $$noise=\sqrt{\frac{\sum_{i=1}^{N}{\left(x_{i}-x\right)}^{2}}{N-1}}$$

where $x_{i}$ is the dataset value at time *i*, x is the average value of the dataset and *N* is the total size of the data. It was found that the lowest noise value (0.0022 m/s^2^) was given by the ADXL354 sensor. In terms of power consumption and noise, the ADXL354 thus seems to be the best accelerometer, but its performance needs to be confirmed experimentally with the vibrating table.

### 3.3. Results Obtained with the Vibrating Table

#### 3.3.1. Signal Processing

**Figure 6:**
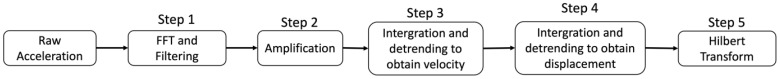
Description of the five steps adopted to extract the displacement time histories from raw acceleration data.

In standard monitoring strategies, pavement health is assessed on the basis of the magnitude of vertical displacements induced by the traffic. As the acceleration data contain noise, double integration over time can lead to loss of resolution and an incapability to detect drifts from the (undamaged) baseline. The signal processing adopted here is based on the work carried out in [18]: for additional details and for a thorough description of the entire processing procedure, readers are referred to the aforementioned paper. In total, the acceleration data are subjected to five different signal processing steps, as shown in Figure 6 and also summarized in [18]. This signal processing is carried out directly on the roadside unit by the Raspberry Pi, using the Python programming language and several libraries like scipy, pandas, matplotlib and numpy. Details on the role of the different filtering steps in obtaining informative time histories of the vertical pavement displacement are described in the following; additional details can be found in [25].

**Figure 7:**
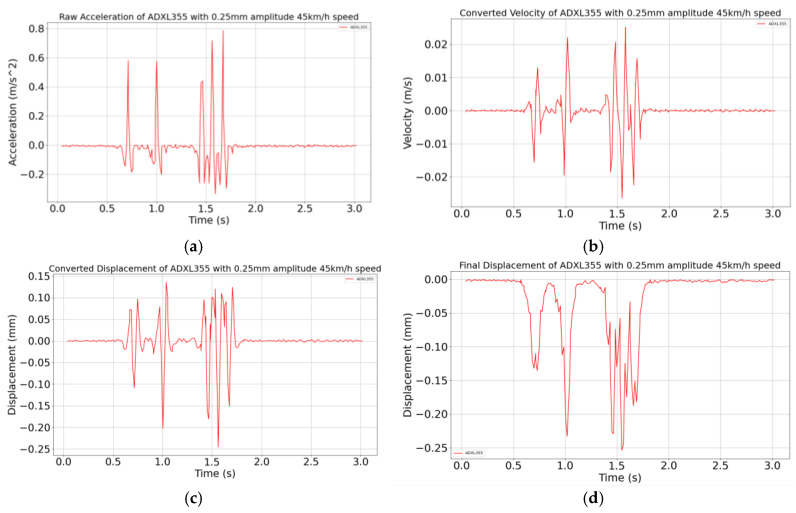
Vibrating table tests: (**a**) example of raw acceleration signal; (**b**) velocity history after the first integration; (**c**) displacement history after the second time integration; (**d**) final displacement history provided by the Hilbert transform.

An example of the raw acceleration data acquired by the MEMS accelerometers is shown in Figure 7a. In any measurement, the recorded acceleration data contain a certain amount of random noise and DC error (drift error) which can be expressed by Formula (2) where *a*(*t*) is acceleration measured with noise, *a*′(*t*) is pure acceleration and *ε* is random noise and DC error.

(2) $$a\left(t\right)=a^{\prime }\left(t\right)+\varepsilon$$

A Fast Fourier Transform is first performed to obtain the frequency spectrum of the raw acceleration signal. Based on the obtained frequency spectrum, the upper and lower cut-off frequencies are selected for the bandpass filter; an appropriate choice of upper cut-off frequencies helps to reduce the high-frequency noise, whereas an appropriate choice of lower cut-off frequency removes the DC component that causes the acceleration signal not to be exactly zero in the absence of motion. In our work, the lower cut-off frequency is 0.2 Hz and the upper cut-off frequency is 40 Hz, as the typical frequency band of truck vibrations extends from 0.5 Hz to 40 Hz. The minimum sampling frequency must comply with Nyquist’s theorem, according to which the sampling frequency of the device must be twice the highest frequency of the waveform. The minimum sampling frequency required is therefore equal to 80 Hz. With the filtering step, the amplitude of the acceleration signal is reduced, and an amplification is adopted to recover the amplitude of the original signal.

Next, time integration is performed to obtain the velocity as shown in Equation (3) while numerical implementation using a python integration program is carried out with the trapezoidal rule presented in Equation (4) where v(t) is the velocity, $v_{0}$ is the initial velocity, $a^{\prime }\left(t_{i}\right)$ is the pure acceleration value at time $t_{i}$, $\varepsilon \left(t_{i}\right)$ is the noise at time $t_{i}$ and Δt is the time interval between the two points ($t_{i+1}$ − $t_{i}$). After integration, it is observed that the signal has an upward trend caused by the noise ε that is integrated and accumulated over time alongside the actual signal as shown in Equation (4). This upward trend should be removed with detrending, resulting in a signal, as shown in Figure 7b;

(3) $$v\left(t\right)=\int [a^{\prime }\left(t\right)+\varepsilon ]\;dt+v_{0}=\int a^{\prime }\left(t\right)dt+\varepsilon t+v_{0}$$

(4) $$v\left(t_{n}\right)\approx v_{0}+\overset{n-1}{\underset{i=0}{\sum }}\frac{\Delta t}{2}\left(a^{\prime }\left(t_{i}\right)+\varepsilon \left(t_{i}\right)+a^{\prime }\left(t_{i+1}\right)+\varepsilon \left(t_{i+1}\right)\right)$$

A second time integration is then performed to obtain the displacement as explained in Equations (5) and (6) where d(t) is the displacement, $d_{0}$ is the initial displacement, $v\left(t_{i}\right)$ velocity value at time $t_{i}$ and $\varepsilon_{v}\left(t_{i}\right)$ is the noise resulting from velocity integration at time $t_{i}.$ As with the first integration, an upward trend is observed because the noise generated by the velocity integration builds up again and detrending is required to obtain the correct displacement recording as observed Figure 7c.

(5) $$d\left(t\right)=\int \left[v\left(t\right)+\varepsilon_{v}\right]\;dt+d_{0}=\int v\left(t\right)dt+\varepsilon_{v}t+v_{0}$$

(6) $$d\left(t_{n}\right)\approx d_{0}+\overset{n-1}{\underset{i=0}{\sum }}\frac{\Delta t}{2}\left(v\left(t_{i}\right)+\varepsilon_{v}\left(t_{i}\right)+v\left(t_{i+1}\right)+\varepsilon_{v}\left(t_{i+1}\right)\right)$$

Since a curve with a positive displacement is observed, an additional signal processing step is necessary with the application of the Hilbert transform [26]. Let us call $x_{r}\left(t\right)$ the real time domain displacement signal with positive displacement depicted in Figure 7c. The Hilbert transform $x_{i}\left(t\right)$ of $x_{r}\left(t\right)$ is calculated with Equation (7). Using $x_{r}\left(t\right)$ and $x_{i}\left(t\right)$, a new analytic signal $x_{c}\left(t\right)$ is generated in Equation (8), the amplitude of which, *E*(*t*), is given by Equation (9). The amplitude is then negatively inverted to obtain the correct displacement value.

(7) $$x_{i}\left(t\right)=\int_{-\infty }^{\infty }\frac{1}{\pi }\;\frac{x_{r}\left(\tau \right)}{\left(t-\tau \right)}d\tau =\frac{1}{\pi }*\;x_{r}\left(t\right)\;$$

(8) $$x_{c}\left(t\right)=x_{r}\left(t\right)+\;jx_{i}\left(t\right)$$

(9) $$E\left(t\right)=\left|x_{c}\left(t\right)\right|=\sqrt{x_{r}{\left(t\right)}^{2}+x_{i}{\left(t\right)}^{2}}\;$$

The upward trend that is removed by detrending means that the signal shape is shifted upwards, which is different from the curve with positive displacement that should not be present and is removed by the Hilbert transform. A typical example of the final result after applying the whole procedure is shown in Figure 7d.

#### 3.3.2. Tests at Varying Displacement Peaks

The laboratory tests were conducted at varying conditions that might be directly related to traffic conditions. In the following, we focus on the results attained by varying the maximum amplitude of the displacement imposed by the vibration table. Such a displacement should, therefore, be considered the target value for the lateral pavement deflection arrived at by way of the signal processing procedure described herein when handling the local accelerations measured by the monitoring device.

**Figure 8:**
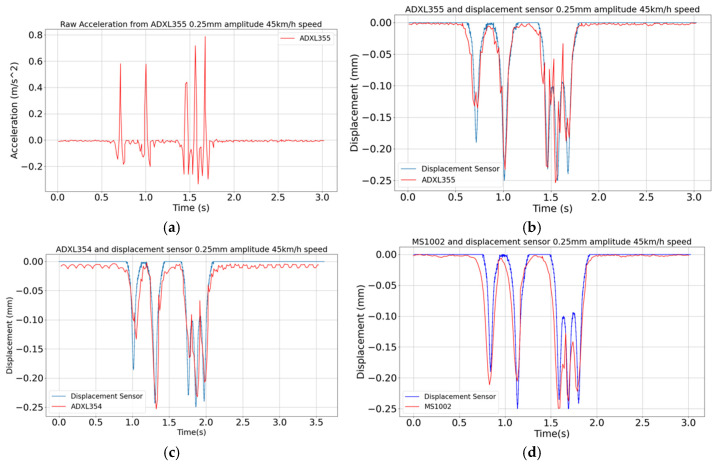
Vibrating table tests featuring a peak displacement of 0.25 mm and a vehicle speed of 45 km/h: (**a**) exemplary raw ADXL355 acceleration signal; (**b**–**d**) displacement histories obtained with the adopted signal processing procedure applied to measurements collected with the three MEMS accelerometers.

The outcomes of the vibration table test are depicted in Figure 8a–d for the case featuring a peak displacement of 0.25 mm and a vehicle speed of 45 km/h. The exemplary raw acceleration signal measured with the ADXL355 sensor is shown in Figure 8a while Figure 8b–d report the displacement histories obtained by processing the measurements collected with the three sensors (ADXL355, ADXL354 and MS1002). Note that the displacement signal obtained with the ADXL354 sensor shows a ripple when no displacement is applied, and the data should be zero, as shown in Figure 8c. This is due to the noise generated by the sensor, which the applied bandpass filter is not fully able to compensate for. The other two transducers, specifically ADXL355 and MS1002, do not show these types of ripples and therefore give better results under these conditions.

**Figure 9:**
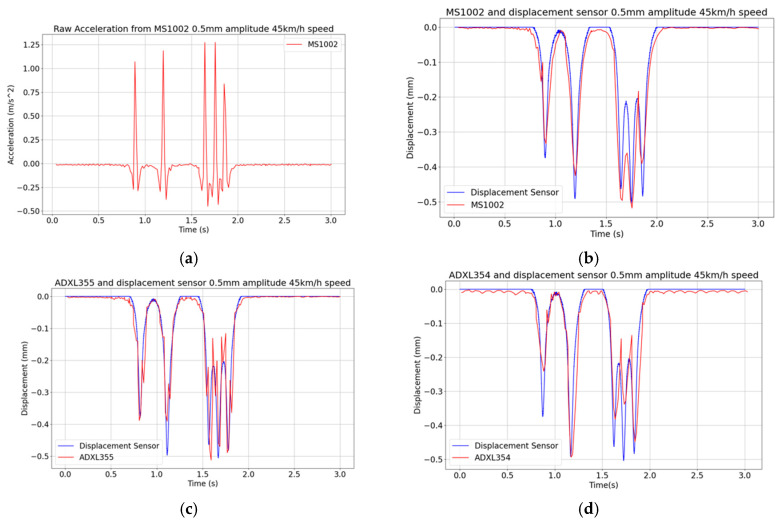
Vibrating table tests featuring a peak displacement of 0.5 mm and a vehicle speed of 45 km/h: (**a**) exemplary raw MS1002 acceleration signal; (**b**–**d**) displacement histories obtained with the adopted signal processing procedure applied to measurements collected with the three MEMS accelerometers.

Figure 9a shows the raw acceleration signal obtained with the MS1002 sensor for the case featuring a peak displacement of 0.5 mm, still with a vehicle speed of 45 km/h. The time histories of displacement obtained with the three sensors are next given in Figure 9b–d. It is shown that the displacement signal supplied by the ADXL354 accelerometers, see Figure 9d, is again characterized by a ripple.

On a purely qualitative basis, the tests at varying displacement peaks confirmed that the signal processing algorithm adopted to convert acceleration into displacement time histories works correctly. It enabled us to obtain the correct shape of the displacement signals, in line with the results available in the literature. Still, imperfections regarding the details of the time histories close to the displacement peaks have to cope with; such imperfections are supposed to be mainly caused by a limited data rate. In future tests featuring a higher data rate, more points will be available, and the peaks are therefore supposed to be better reconstructed. The main aim of these preliminary tests is not only to validate the signal processing algorithm, but also comparatively assess the performance of the three tested accelerometers with the reference sensor integrated into the vibrating table. Due to the aforementioned inaccuracies, an error indicator for the prediction of the shape of the signal is defined by taking into account the complete converted displacement signals, according to the following formula:

(10) $$EI=\frac{1}{N}\;\overset{N}{\underset{i=1}{\sum }}\frac{\left|Ref\;sensor\left(i\right)-sensor\;value\left(i\right)\right|}{Max\left(Ref\;sensor\right)}\times 100\;$$

where *EI* is the error indicator; *N* is the size of the dataset; *Ref sensor*(*i*) is the vector of reference sensor data; *sensor value*(*i*) is instead the vector of the sensed data; and *Max*(*Ref sensor*) is the maximum displacement value measured by the reference sensor. Another error indicator is also suggested to evaluate the prediction accuracy of the five displacement peaks observed with the three accelerometers, according to the following:

(11) $$Peak\;EI=\frac{1}{5}\;\overset{5}{\underset{i=1}{\sum }}\frac{\left|Ref\;sensor\;peak\left(i\right)-sensor\;peak\left(i\right)\right|}{Max\left(Ref\;sensor\;peak\right)}\times 100\;$$

where *Peak EI* is the peak error indicator and, similarly to Equation (10), *Ref sensor peak*(*i*) is the vector of reference sensor peak data; *sensor peak*(*i*) is the vector of sensed peak data; and *Max(Ref sensor peak)* is the maximum displacement peak value measured by the reference sensor among the five peaks. The data collected in Table 2 show that the error indicator is less than 5% for a displacement of 0.25 mm, regardless of the sensor adopted, while it is around 8–9% for a displacement of 0.5 mm. While MS1002 and ADXL355 show a *Peak EI* value of around 4–6% for 0.25 mm and 0.5 mm, it is more than 10% for ADXL354. The superiority of MS1002 and ADXL355 over ADXL354 is less obvious in relation to *EI*: for all the sensors, *EI* deteriorates with an increasing displacement. In conclusion, the *EI* and *Peak EI* values indicate an acceptable level of accuracy for the whole procedure in terms of predicted displacement shape and peak, given that the relative variation in these values over time, rather than their absolute values, is the key information used for monitoring pavement evolution and potential degradation.

#### 3.3.3. Tests at Varying Vehicle Speed

**Figure 10:**
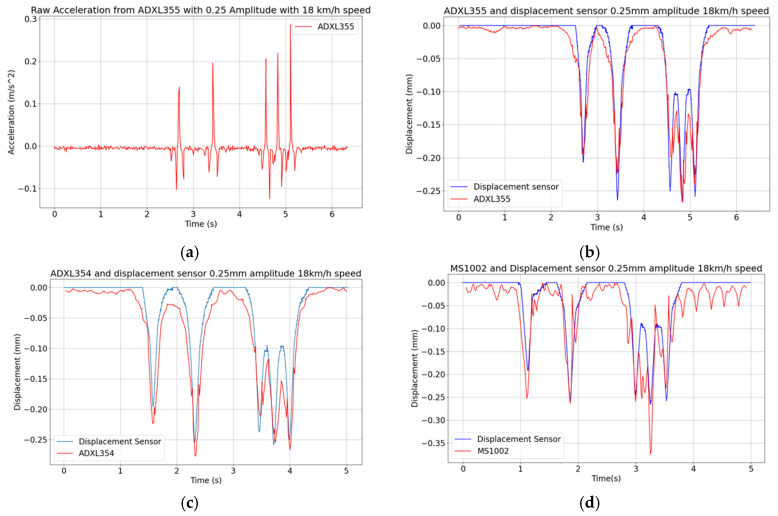
Vibrating table tests featuring a peak displacement of 0.25 mm and a vehicle speed of 18 km/h: (**a**) raw ADXL355 acceleration signal; (**b**–**d**) displacement histories obtained with measurements collected with the three MEMS accelerometers.

**Figure 11:**
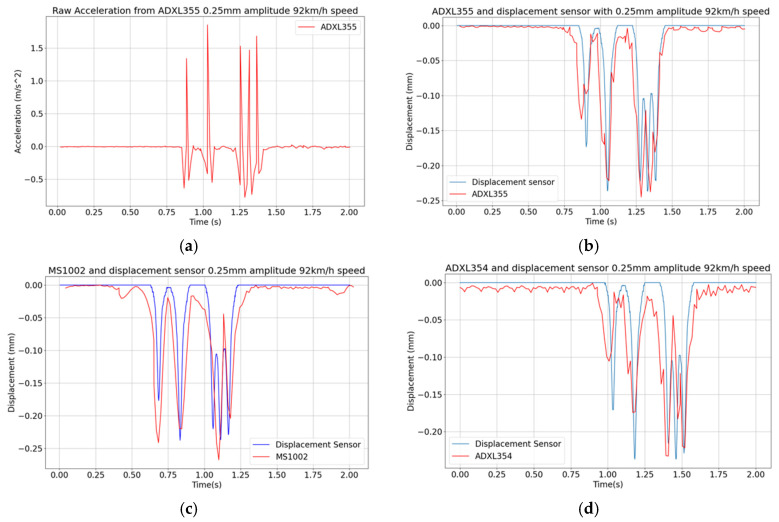
Vibrating table tests featuring a peak displacement of 0.25 mm and a vehicle speed of 92 km/h: (**a**) raw ADXL355 acceleration signal; (**b**–**d**) displacement histories obtained with measurements collected with the three MEMS accelerometers.

Keeping the displacement amplitude of 0.25 mm as a reference, two additional vehicle speeds of 18 km/h and 92 km/h are considered, leading to the results reported in Figure 10 and Figure 11. It can be seen that at lower speeds, like, e.g., at the reported 18 km/h, noise indeed affects more the results and spoils somehow the signal. As far as the MS1002 sensor is concerned, even after applying the bandpass filter the results still show many ripples. The maximum peak is also higher than the expected one measured by the reference sensor.

In relation to the data obtained with the high vehicle speed of 92 km/h, in Figure 11a–d it can be seen that the ADXL355 provides the same displacement pattern as the reference sensor, while the other two sensors lose important features like the displacement peaks, due to a slow data throughput. On the basis of these laboratory tests, it can be concluded that the minimum acquisition rate required to capture the effects on the pavement of a vehicle at higher speed is at least 80 Hz, which is allowed only by ADXL355 among the selected accelerometers. The error indicators are reported in Table 3. *EI* is higher at the lowest speed 18 km/h for all three sensors as more noise is captured. For *Peak EI*, the ADXL354 and ADXL355 give an error value of less than 10% while the MS1002 sensor gives 16.6% at 18 km/h (several peaks away from peak values of the reference) and 22.2% at 92 km/h (one displacement peak missing due to a too slow data rate). It can be seen that the MS1002 sensor gives the highest *EI* and *Peak EI* for all tests, while theADXL355 has the most accurate *Peak EI* for both speeds. In the light of the previous results, it appears that the ADXL355 seems to be the best candidate if we consider that the deviation from the reference sensor signal is important. On this basis, the ADXL355 is therefore used in the in situ tests reported in the following section.

## 4. Preliminary Tests on a Real Pavement

### 4.1. Experimental Setup

**Figure 12:**
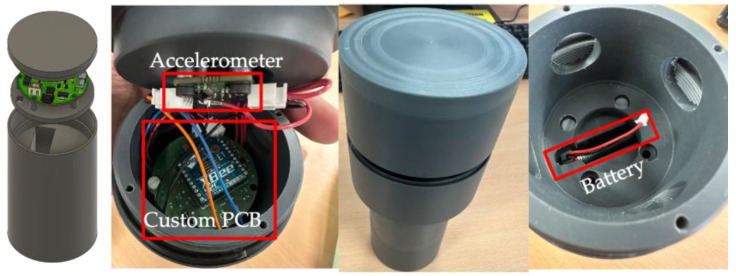
Designed and fabricated PVC packaging, and assembly of the embedded unit.

Once laboratory experiments have produced promising results in terms of sensors and wireless connectivity, the next step to report is a real test on a road. A package has first been designed and manufactured to encapsulate all the electronics required for the pavement test. The packaging for the on-board unit, to be embedded into the pavement test track, has been designed using the Autodesk Inventor 2023.3 software and fabricated using a machine-tooled PVC cylinder; see Figure 12. On the basis of the better performances reported in the former laboratory tests, the ADXL355 accelerometer is selected. As shown in the pictures, the sensor is connected to the on-board unit printed circuit board, which is fixed to the lid of the PVC cylinder. The PCB is attached to the top of a 3D printed stand with a screw, then placed inside the package. The battery is placed underneath and connected to the PCB via the battery connector.

**Figure 13:**
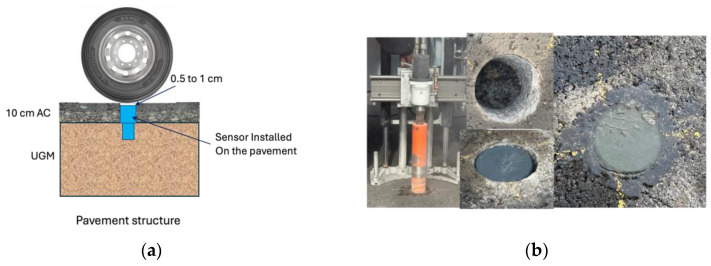
(**a**) Device installation scheme; (**b**) installation of the device in the pavement.

Pavement tests with the developed system were carried out on the test track of the accelerated pavement testing facility of Gustave Eiffel University in Nantes. There are several steps involved in installing the on-board unit in the roadway. A diagram of how to install the sensor in the roadway is shown in Figure 13a. The first step is to drill a hole in the pavement, as shown in Figure 13b. After drilling the hole in the pavement, the device is inserted into the hole less than 1 cm below the pavement surface. Next, the hole containing the device is filled with resin to bond, encapsulate and fix the device to the pavement. The test can then be carried out 24 h after installation, while waiting for the resin to dry. Resin is important to protect the device from humidity, changing environmental conditions and water spreading inside the pavement. The device proved to operate reliably in real-life scenarios when embedded in the roadway, even in environmental conditions characterized by high temperatures of up to 46 °C.

**Figure 14:**
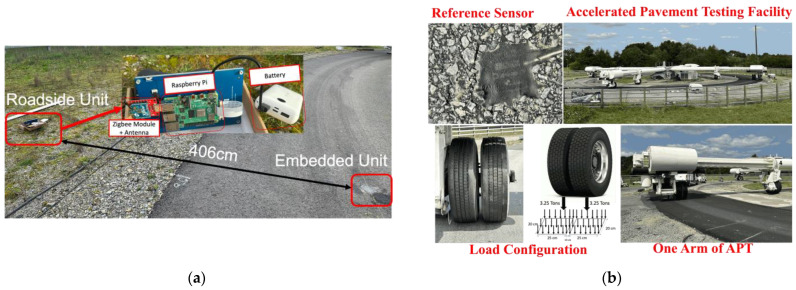
(**a**) Position of the roadside unit on the test track; (**b**) accelerated pavement testing setup.

In this experimental campaign, the accelerated pavement testing facility with a fatigue carousel is used to carry out the evaluation test (see Figure 14b). A Silicon Design reference 21102-002 accelerometer is also installed in the same pavement section and connected separately to the roadside unit using a cable with a 1200 Hz data rate. The load configuration is a double-wheeled truck axle, with each axle having a square contact surface with the pavement of 25 cm × 20 cm and a weight of 3.25 tons. The load rotates continuously to mimic a vehicle moving at a speed of 36 Km/h. During the experiment, one wheel is set to pass directly over the on-board unit.

### 4.2. Test Results

**Figure 15:**
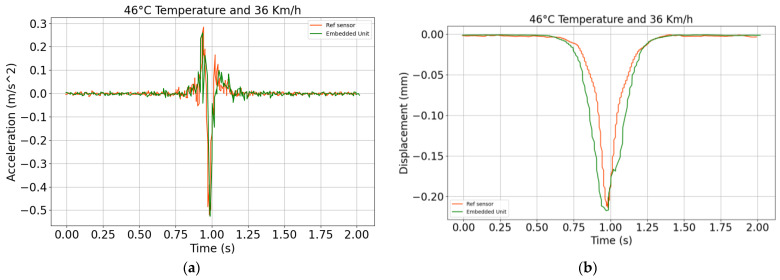
(**a**) Raw acceleration, and (**b**) displacement time history obtained with the reported signal processing strategy.

Figure 15a shows the raw accelerometer signals collected during the test with the on-board unit (green line) and the reference sensor (red line). It can be observed that the two signals are slightly different in amplitude; the reference sensor, due to its 1200 Hz data rate, is able to capture higher frequency oscillations in the pavement, although these are of a limited importance as far as the relevant health is a concern. The adopted accelerometer, characterized by a 130 Hz data rate, proves sufficient to comply with Nyquist sampling theorem and sense the pavement vibration signal. The raw acceleration signals are next converted into displacement, and the results are reported in Figure 15b. The two signals to compare show a displacement peak of 0.22 mm in the case of the on-board unit, and of 0.21 mm in the case of the reference sensor. The discrepancy between the two datasets is again evaluated by means of the *EI* and *Peak EI* metrics, leading to *EI* = 4.2% and *Peak EI* = 5.4%. This further result shows that the sensor is capable to perform displacement monitoring in the pavement, with a good accuracy both in terms of *EI* and *Peak EI* values.

Further tests are currently underway, or will be carried out in the near future, to analyze various parameters such as load position and weight, speed and different environmental conditions such as road surface temperature.

## 5. Comparison with Setups Available in the Literature

A comparison of the proposed system with other configurations available in the literature is finally presented in Table 4. Compared with the devices presented in [1,4], our solution offers advantages in terms of cost and fully wireless capability. Another advantage is the computational capacity at the edge of the roadside unit, since the conversion of acceleration signals into displacement signals is performed directly by the unit itself. On the other hand, the main problem to be solved in the future is linked to the limited lifespan of the on-board unit.

## 6. Conclusions

A wireless sensor network for pavement monitoring based on the Zigbee protocol has been developed to monitor the health of road pavements, with the goal of preventing or at least reducing possible accidents induced by the diffuse damage patterns. The Zigbee protocol has been chosen in place of other wireless communication ones because of its lower power consumption in the transmission mode. The aim is, therefore, to extend the lifetime of the monitoring systems as much as possible, and avoid expensive and time-consuming replacements of the devices buried under the pavement. A signal processing algorithm has been implemented to convert the acceleration signals recorded by the MEMS accelerometer embedded into the developed device into displacement time histories that can be better liked to the pavement’s physical properties. The signal processing has yielded promising results, although some imperfections still need to be handled, as can be seen in comparison between the converted displacement evolution and the reference measurements taken during the laboratory tests with a vibrating table. The developed system has also been tested in the field.

According to the results of the laboratory tests, the ADXL354 accelerometer has displayed the lowest energy consumption, and would therefore allow for the longest lifetime of the monitoring system. On the other hand, the ADXL355 accelerometer has performed better in the vibrating table tests, with a lower level of noise that can affect the measurements. The ADXL355 accelerometer was therefore chosen for the field tests.

The results of the field tests have shown that the raw acceleration signals obtained from the measurements have a good shape and amplitude when compared with the acceleration signal obtained with a reference sensor at the testing facility. The displacement signals obtained with the processing procedure have resulted in *EI* and *Peak EI* error measures smaller than 10%. While the results of the laboratory and real pavement tests are deemed promising, there are still limitations linked to the device lifetime that need to be solved in the near future.

In terms of costs, that for building the on-board unit is around 100 euros while that for the entire system comprising the roadside unit is USD 215. This overall cost is therefore lower than the costs of the other systems presented in [1].

Further field tests are going to be carried out at the accelerated pavement testing facility, featuring different load weights, speeds and lateral load positions with respect to sensor positioning. A test on a real highway with a truck load will also be carried out in the future. In addition, an automatic wake-up and stand-by strategy is going to be implemented when vehicles pass by, to save energy and ensure that the system is always capable of collecting information on the pavement’s state, instead of waiting for the roadside unit to transmit a start-up or stand-by command given by an operator when the monitoring is needed. Future research will focus on developing an automatic wake-up mechanism to reduce power consumption and on the development of distributed measurement systems with numerous on-board units.

## Figures and Tables

**Table 1:** Sensor characteristics obtained with the vibrating pot test.

Accelerometer Type	ADXL355	ADXL354	MS1002
Output	Digital Output	Analog Output	Differential Analog Output
Sensitivity	256 LSB/g [24]	384 mV/g	1340 mV/g
Power Consumption	201.0 μA	155.2 μA	12.11 mA
Communication method	I2C	Analog Input of ADS1115 ADC	Analog Input of ADS1115 ADC
Sampling Frequency	80 Hz	70 Hz	70 Hz
Noise	0.0024 m/s^2^	0.0022 m/s^2^	0.0076 m/s^2^

**Table 2:** Vibrating table tests: values of the error indicators for 0.25 mm and 0.5 mm displacement at a 45 km/h speed.

Sensor	0.25 mm Peak		0.5 mm Peak	
	*EI*	*Peak EI*	*EI*	*Peak EI*
MS1002A	3.3%	4.4%	8.7%	6.4%
ADXL354	4.5%	12.8%	8.9%	15.5%
ADXL355	3.8%	5.6%	7.7%	4.6%

**Table 3:** Vibrating table tests: values of the error indicators for 0.25 mm displacement at 18 km/h and 92 km/h vehicle speeds.

Sensor	18 km/h Speed		92 km/h Speed	
	*EI*	*Peak EI*	*EI*	*Peak EI*
MS1002	14.2%	16.6%	13.1%	22.2%
ADXL354	10.6%	7.7%	9.4%	8.0%
ADXL355	12.0%	3.5%	8.1%	7.2%

**Table 4:** Comparison between the proposed setup and those available in the literature.

Parameters	Proposed System	[4]	[1]
Cost	USD 215	USD 19,140	USD 23,500
Sensor Communication with acquisition unit	Zigbee Xbee S2C wireless communication module	Cable	Wireless RF transceiver CC2420
Parameters Measured	Acceleration from pavement vibration converted into pavement displacement	Asphalt Strain Response	Acceleration from pavement vibration
Sensor Used	ADXL355	Vertical and Horizontal Strain Gage, and Load Cell	MS9002.D MEMS Accelerometers
Acquisition Unit	Raspberry Pi 4	V-Link Wireless Voltage Nodes	Wireless access point
Advantages	Offering the Lowest cost. Fully wireless for sensor data acquisition. Signal Processing happens in the roadside unit.	Wireless communication with the base station	High data rate with 512 Hz. Better lifetime and power consumption. Wireless firmware update.
Drawbacks	Battery powered with limited device lifetime.	Cables to connect the sensor to the roadside unit. Cost is still high.	High cost. Data extraction required to process data.

## Data Availability

The data that support the findings of this study are available from the corresponding author upon reasonable request.
